# Upgulation of lncRNA GASL1 inhibits atherosclerosis by regulating miR-106a/LKB1 axis

**DOI:** 10.1186/s12872-023-03038-9

**Published:** 2023-01-10

**Authors:** Xueqi Rui, Xinning Wu, Zheyi Rong, Zipeng Wang

**Affiliations:** 1 Department of Cardiovascular Medicine, Liyang People’s Hospital, Liyang, 213399 China; 2 Department of Cardiovascular Medicine, People’s Hospital of Rizhao, No. 126 Tai’an Road, Donggang District, Rizhao, 276827 China; 3 Department of Cardiovascular Medicine, Renhe Hospital, Baoshan District, Shanghai, 201900 China; 4 Department of Neurology, Huai’an Second People’s Hospital, The Affiliated Huai’an Hospital of Xuzhou Medical University, No. 62, Huaihai South Road, Qingjiangpu District, Huai’an, 223000 China

**Keywords:** GASL1/miR-106a/LKB1, Atherosclerosis, Diagnosis, Viability, Inflammation

## Abstract

**Background:**

Atherosclerosis (AS) is a common frequently-occurring disease in the clinic and a serious threat to human health. This research aimed to explore the value between GASL1 and AS.

**Methods:**

The expression and values of GASL1 in AS patients were revealed by qRT-PCR and ROC curve. The HUVEC cells were induced by ox-LDL to construct in-vitro models. Cell viability was detected by MTT assay, and apoptosis was detected by flow cytometry. The inflammatory situation was reflected by the ELISA assay. Double luciferase reporter gene assay verified the regulatory relationship between GASL1 and miR-106a, miR-106a and LKB1.

**Results:**

The levels of GASL1 was lower in AS group than those in control group. The value of GASL1 in predicting AS patients was also tested by the ROC curve. After HUVEC cells were induced by ox-LDL, the levels of GASL1 and LKB1 decreased significantly, while the level of miR-106a increased significantly. Upregulation of LKB1 reversed the effect of upregulation of GASL1 on viability, apoptosis, and inflammation of HUVEC cells induced by ox-LDL.

**Conclusion:**

Overexpression of GASL1 might suppress ox-LDL-induced HUVEC cell viability, apoptosis, and inflammation by regulating miR-106a/LKB1 axis.

## Introduction

Atherosclerotic cardiovascular and cerebrovascular diseases represented by stroke and coronary heart disease are involved in the majority of deaths worldwide [1]. Atherosclerosis (AS) is a chronic progressive disease, and the gradual increase of plaques can cause carotid stenosis, leading to the insufficient blood supply to the brain tissue [2]. Although the degree of luminal stenosis caused by the plaque is not high, the nature of the plaque itself has changed, such as the formation of a large necrotic core, a thin fibrous cap, inflammatory response, and surface ulcers, and intra-plaque hemorrhage [3]. Multiple imaging and histological studies have confirmed that such plaques are “unstable plaques”, which are prone to rupture and fall off, triggering acute cerebrovascular embolism [4]. Although the progress of carotid atherosclerotic plaques and the formation of unstable plaques are related to traditional factors such as hypertension, hyperlipidemia, and smoking, there are still many patients with carotid stenosis who do not have traditional risk factors and suddenly suffer from transient amaurosis, TIA, stroke and other acute cerebral ischemia symptoms [5, 6]. Therefore, further exploration of biomarkers for AS contributes to the treatment of such patients.

LncRNA is a kind of non-coding RNA with a highly conserved sequence, which does not have an open reading frame that encodes protein [7]. Increasing evidence shows that lncRNA regulation is closely related to human diseases (tumors, cardiovascular diseases, etc.) and plays an important role in AS. LncRNA TUG1, AK098656, TRPV1, GAS5, and Inc-Ang362 are implicated in hypertension-related vascular remodeling, and H19, TUG1, and UCA1 are involved in AS [8]. LncRNAKCNQ1OT1 is down-regulated in VSMCs treated with platelet-derived growth factor, while up-regulated KCNQ1OT1 expression upregulates the expression of nuclear transcription factor kappa⁃Ba through the interaction with miR⁃221, thus inhibiting the inflammation and proliferation of VSMCs and alleviating the intimal hyperplasia [9]. LncRNA NORAD mitigates endothelial cell senescence and inhibits cell apoptosis, and atherosclerosis via interacting with NF-κB and p53-p21 axis [10]. LncRNA GASL1 is a common non-coding RNA, which is widely researched. GASL1 is downregulated in chronic heart failure, and upregulation of GASL1 may ameliorate this disease by suppressing cardiomyocyte apoptosis via interacting with TGF-β1 [11]. Chronic heart failure (CHF) is a serious manifestation of various cardiovascular diseases, including AS [12]. However, unclear underlying pathology between GASL1 and AS exists.

Considering the aforementioned researches, it was hypothesized that the expression of GASL1 was lessened and it might participate in the pathology of AS. For this hypothesis, this study purposed to assess the expression of GASL1 in AS patients and estimate its function in AS cell models. In the current investigation, collective patients and control individuals were applied to assess the expression of GASL1 and evaluate the diagnostic role of GASL1. In addition, the potential interactions between GASL1 and AS were revealed by constructing cell models via oxidized low-density lipoprotein (ox-LDL). The impacts of GASL1 on cell viability, apoptosis, and inflammation were tested. The targeted ceRNA and gene were further verified.

## Materials and methods

### Patients and sample collection

A total of 87 patients with carotid atherosclerosis treated in People’s Hospital of Rizhao were selected as the atherosclerosis group. Another 87 people without carotid atherosclerosis who were examined in our hospital at the same time were selected as the control group. Carotid intima-media thickness (CIMT) of 0.9–1.2 mm was the standard for all patients. Exclusion criteria included acute and chronic infections; a definite history of ischemic stroke or cerebral hemorrhage; severe dysfunction of heart, liver, kidney, and other organs; malignant tumor; recent surgical history; history of craniocerebral trauma.

This study was performed in line with the principles of the Declaration of Helsinki. All the research subjects and their families were informed, signed the informed consent form, and approved by the ethics committee of People’s Hospital of Rizhao.

### Cell incubation and transfection

Obtained HUVECs were from the Chinese Academy of Sciences Cell Bank (Shanghai, China). Resuscitated HUVECs were added into DMEM medium containing 10% FBS, which was cultured in an incubator with 37 °C, 5% CO_2_ volume fraction, and 97% humidity. Logarithmic HUVECs were inoculated in a 6-well plate and were treated with various concentrations (0, 25, 50, 100 µg/ml) of ox-LDL for 24 h. With lipofectamine™ 2000 kit, pCDNA-GASL1, pCDNA-negative control (NC), miR-106a-mimic, miR-106a-inhibitor, miR-106a-NC were transfected to HUVECs.

### Quantitative real-time PCR (qRT-PCR)

The patient’s serum was taken out and dissolved at room temperature. After each case was shaken and centrifuged gently, 200 µl of serum was drawn into clean EP tubes, and 200 µl of lysis reagent was supplemented to each tube. The samples were mixed with sufficient shaking and placed at room temperature for 3 min. Buffer RCA, chloroform, and absolute ethanol were added to the solution. The mixture was centrifuged at room temperature, and the adsorption column was placed in a new EP tube. DEPC water was added to the adsorption column to dissolve the total RNA. And the purity and concentration of the mRNA samples were determined on a spectrophotometer. The kit applied was from Sangon (Shanghai, China). cDNA was synthesized by cDNA synthesis kit (Applied Biosystems, Foster City, USA) or miR CURY LNA Universal RT microRNA PCR kit (Exiqon, Denmark). 2X SG fast qPCR mix from Sangon was used to assess the relative expression on ABI 7500 system. The relative expressions of GASL1 and LKB1 mRNA to GAPDH and miR-106a to U6 were calculated by the 2^−∆∆Ct^ method.

### Detection of cell proliferation by MTT

200 µ l HUVECs or transfected cells were inoculated in a 96-well plate and cultured for 4 h, then abandoned the medium. Then, 20 µl MTT (Sigma, St Louis, USA) was added to each hole and incubated for 4 h. The culture medium was abandoned, 150 µl dimethyl sulfoxide was supplemented to each hole, and the absorbance was tested at 490 nm.

### Detection of apoptosis by flow cytometry

The cells were inoculated and cultured, and the cells of each group were collected. After washing with PBS, add 500 µl binding buffer to resuscitate the cells. Add 10 µl annexin V-fluorescein isothiocyanate (Promega, Madison, USA) and incubate for 10 min at room temperature without light. 5 µl propidium iodide was added for 5 min, and the mixture was detected by flow cytometry.

### Enzyme-linked immunosorbent assay (ELISA)

According to the instructions of the kit (Cloud-Clone Corp, Houston, USA), the supernatants of the collected cells were detected for IL-1 β and IL-6 The supernatant was centrifuged at 4 °C and centrifuged for 2000 g 20 min. The supernatant was further diluted, and the concentration gradient standard was prepared by diluting the standard sample of 1ng/ml 5 times. The tested samples and standard samples were added to the ELISA micropores coated with antibodies, A solution, and B solution in turn. Then, 90 µl TMB substrate was added to each hole to incubate 25 min at 37 °C, and the termination solution was added. SpectroMaxM5 was used to measure and analyze immediately.

### Luciferase activity report assay

The nucleotide sequence of GASL1 containing miR-106a binding site was synthesized by Shanghai GenePharma Co. (China) and inserted into pGL3-Promoter plasmid vector to construct GASL1 wild-type recombinant plasmid (GASL1-WT). The binding site was mutated by site-directed mutagenesis and inserted into pGL3-Pro-moter vectors to establish GASL1mutant recombinant plasmid (GASL1-MUT). GASL1-WT and GASL1-MUT, miR-106a NC, miR-106a mimic, and miR-106a inhibitor were co-transfected with LipofectamineTM 2000 kit respectively. The luciferase activity was detected by the double luciferase activity detection kit (Yeason, Shanghai, China), and the luciferase activity was expressed by the fluorescence intensity ratio of firefly and renilla.

### Statistical method

SPSS20.0 and GraphPad statistical software were used for data processing. The measurement data are described by x ± s or number. The independent sample t-test and χ^2^ test were used for the comparison between the two groups, and the comparison among groups was described by one-way ANOVA. The receiver operating characteristic (ROC) curve was provided in this study to evaluate the predictive value of GASL1. The clinical risk factors were estimated by the binomial logistic regression analysis. The difference was statistically significant (*P* < 0.05).

## Results

### Baseline clinicopathological features of all participants

AS group included 46 males and 41 females, with an average of 60.07 ± 12.65 years. There was no significant difference in sex and age between the two groups (Table 1, all *P* > 0.05), so they were comparable. In addition, there was no significant difference in body mass index, high-density lipoprotein, low-density lipoprotein, diabetes, and hypertension between the two groups (Table 1, all *P* > 0.05). The levels of C-reactive protein and carotid intima-media thickness in the AS group were significantly higher than those in the control group (Table 1, all *P* < 0.001).

**Table 1:** Clinical baseline data of the study subjects

Parameter	Control (n = 87)	Atherosclerosis (n = 87)	*P* values
Age, years	59.93 ± 14.19	60.07 ± 12.65	0.946
BMI, kg/m^2^	21.65 ± 2.96	22.42 ± 2.97	0.088
Sex, n			0.544
Male	42	46	
Female	45	41	
LDL-C, mg/dl	121.29 ± 10.93	123.38 ± 9.80	0.186
HDL-C, mg/dl	49.83 ± 4.93	48.74 ± 5.35	0.167
CRP, mg/l	4.68 ± 0.96	9.43 ± 1.09	< 0.001
CIMT, mm	0.55 ± 0.15	1.06 ± 0.08	< 0.001
Diabetes, n			0.280
Yes	32	39	
No	55	48	
Hypertension, n			0.093
Yes	43	54	
No	44	33	

*BMI* body-mass index, *HDL-C* high-density lipoprotein, *LDL-C* low density lipoprotein, *CRP* C-reactive protein, *CIMT* carotid intima-media thickness

### Decreased expression of GASL1 and its predictive role

**Fig. 1:**
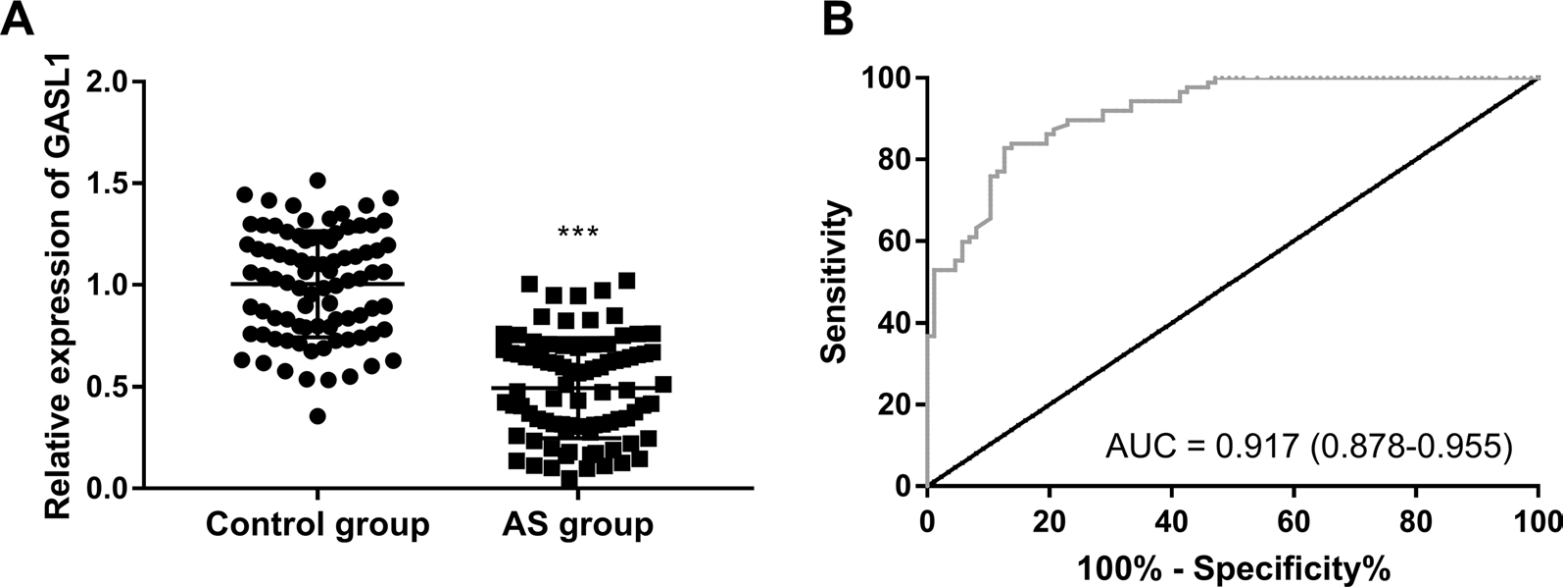
The expression of GASL1 in patients with AS. **A** The decreased GASL1 was found in patients with AS. **B** GASL1 might be a diagnostic biomarker in patients with AS. ***P < 0.001

The expression of GASL1 in patients with AS was lower than that in the control group (Fig. 1A, P < 0.001). The value of GASL1 in predicting the occurrence of AS was evaluated by the ROC curve. As shown in Fig. 1B, the AUC was 0.917 (95% CI 0.878–0.955) with a sensitivity of 0.828, and a specificity of 0.874.

Taken the abnormal expression of GASL1 in patients with AS, a binomial regression analysis was performed to estimate whether GASL1 was a risk variable in the occurrence of AS. As shown in Table 2, the relative GASL1 expression (OR 0.189, 95% CI 0.043–0.823, *P* = 0.026) and CIMT (OR 4.404, 95% CI 1.044–18.582, *P* = 0.044) were both risk parameters for AS patients.

**Table 2:** Binomial logistic regression analysis of clinical risk factors

Parameter	OR values	95% CI		*P* values
		Lower	Upper	
Age, years	1.536	0.572	4.122	0.395
BMI, kg/m^2^	1.314	0.692	2.495	0.404
Sex, n	0.918	0.487	1.731	0.792
LDL-C, mg/dl	0.962	0.506	1.829	0.905
HDL-C, mg/dl	0.721	0.381	1.364	0.315
CRP, mg/l	1.722	0.913	3.249	0.093
CIMT, mm	4.404	1.044	18.584	0.044
Diabetes, n	0.728	0.385	1.378	0.329
Hypertension, n	0.454	0.204	1.012	0.054
Relative GASL1 expression	0.189	0.043	0.823	0.026

*BMI* body-mass index, *HDL-C* high-density lipoprotein, *LDL-C* low density lipoprotein, *CRP* C-reactive protein, *CIMT* carotid intima-media thickness

### GASL1 recovered the damage caused by ox-LDL

**Fig. 2:**
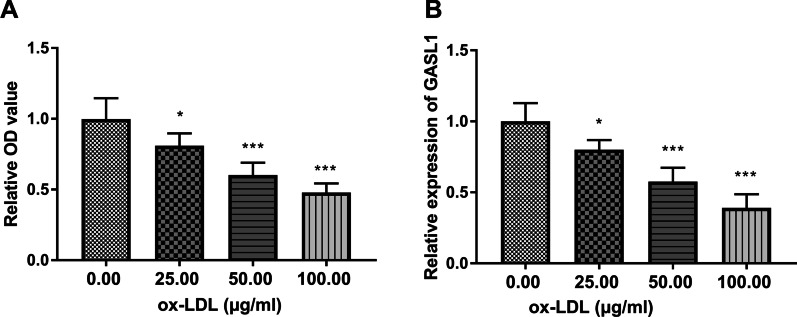
Functions of ox-LDL in HUVECs. **A** The gradually decreased cell viability of HUVECs was caused by ox-LDL. **B** The expression of GASL1 was elevated by ox-LDL. *P < 0.05, ***P < 0.001

The survival rate of HUVECs in ox-LDL group was gradually lessened when compared with the no ox-LDL group (Fig. 2A, P < 0.05). Meanwhile, the expression of GASL1 was decreased in HUVES cells treated with ox-LDL (Fig. 2B, P < 0.05). Considering that 50 µg/ml of ox-LDL could significantly decrease the cell viability and GASL1 expression, 50 µg/ml was taken as the following treatment concentration.

**Fig. 3:**
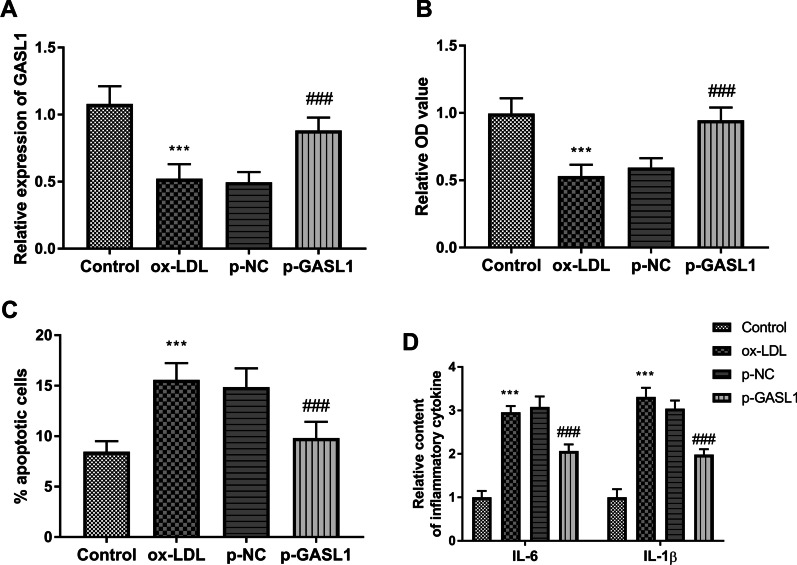
Roles of GASL1 in HUVECs. **A** The transfection of p-GASL1 changed the inhibition of GASL1 in ox-LDL group. **B** GASL1 ameliorated the abnormal cell viability led by ox-LDL. **C** Highly expressed GASL1 decreased the percentage of apoptotic cells in the ox-LDL group. **D** Elevated expression of inflammatory cytokines was found in ox-LDL group and inhibited in the p-GASL1 group. ***P < 0.001, relative to control group; ^###^*P* < 0.001, relative to ox-LDL group

As depicted in Fig. 3A, the expression of GASL1 was decreased in the ox-LDL group and partially recovered in the p-GASL1 group (*P* < 0.001). Moreover, the balance of viability and apoptosis of HUVECs were also damaged by ox-LDL, while the overexpression of GASL1 inhibited these impacts (Fig. 3B, C, P < 0.001). The inflammatory response arose by the treatment of ox-LDL in HUVECs, whereas the increased GASL1 suppressed the abnormal inflammatory situation (Fig. 3D, P < 0.001).

### The ceRNA targeting GASL1

**Fig. 4:**
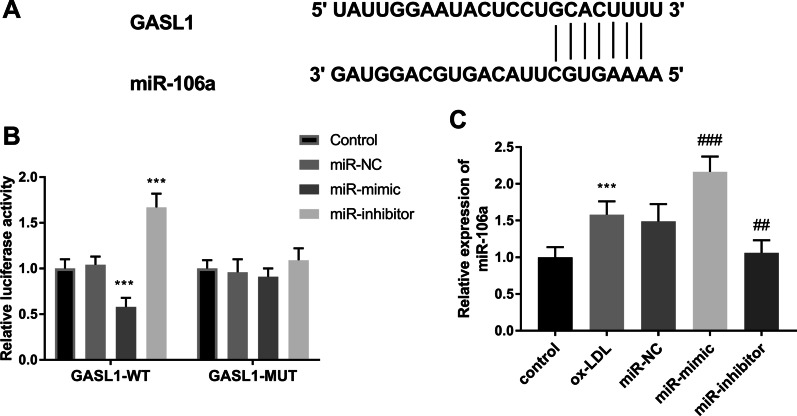
MiR-106a was a sponge of GASL1. **A** The putative binding bases between GASL1 and miR-106a. **B** Luciferase report assay documented the targeted relationship between GASL1 and miR-106a. **C** The expression of miR-106a was increased in ox-LDL group. ***P < 0.001, relative to control group; ^##^*P* < 0.01, ^###^*P* < 0.001, relative to ox-LDL group

The putative targeting bases between miR-106a and GASL1 were shown in Fig. 4A, indicating the targeted interaction between them. In addition, this correction between miR-106a and GASL1 was identified by the luciferase activity report, which documented that the luciferase activity was inhibited by the increased miR-106a expression and elevated by the decreased miR-106a levels in the GASL1-WT group (Fig. 4B, P < 0.001). The expression of miR-106a was diminished in the ox-LDL group compared with control cells (Fig. 4C, P < 0.001). The transfection of miR-106a mimics further aggravated the increment of miR-106a, and the miR-106a inhibitors led to the absence of mIR-106a expression (Fig. 4C, P < 0.01).

### miR-106a mediated the impacts of GASL1 on HUVEC

**Fig. 5:**
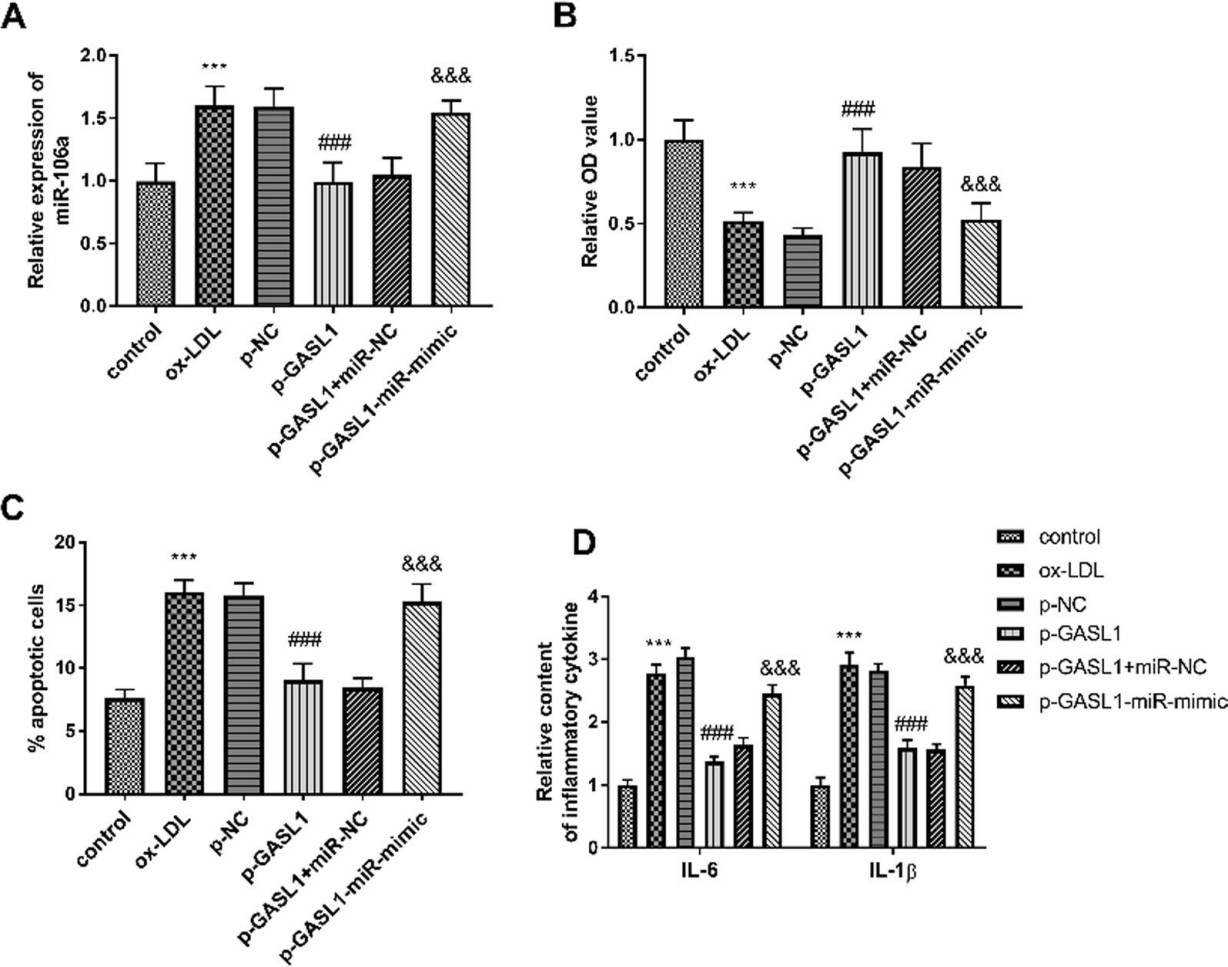
MiR-106a mediated the function of GASL1. **A** The expression of miR-106a was regulated by GASL1. **B–D** GASL1 exerted function on cell viability, apoptosis, and inflammation by sponging miR-106a. ***P < 0.001, relative to control group; ###*P* < 0.001, relative to ox-LDL group; ^&&&^*P* < 0.001, relative to p-GASL1 group

As corroborated in Fig. 5A, the highly expressed in the ox-LDL group was inhibited by enforced GASL1 levels, and this influence was ameliorated by the transfection of miR-106a mimics (*P* < 0.001). The ascended miR-106a expression also destroyed the beneficial impacts of GASL1 on cell viability, apoptosis, and inflammatory dysfunction (Fig. 5B–D, P < 0.001), indicating the interconnection between miR-106a and GASL1.

### LKB1 serves as a target gene of miR-106a

**Fig. 6:**
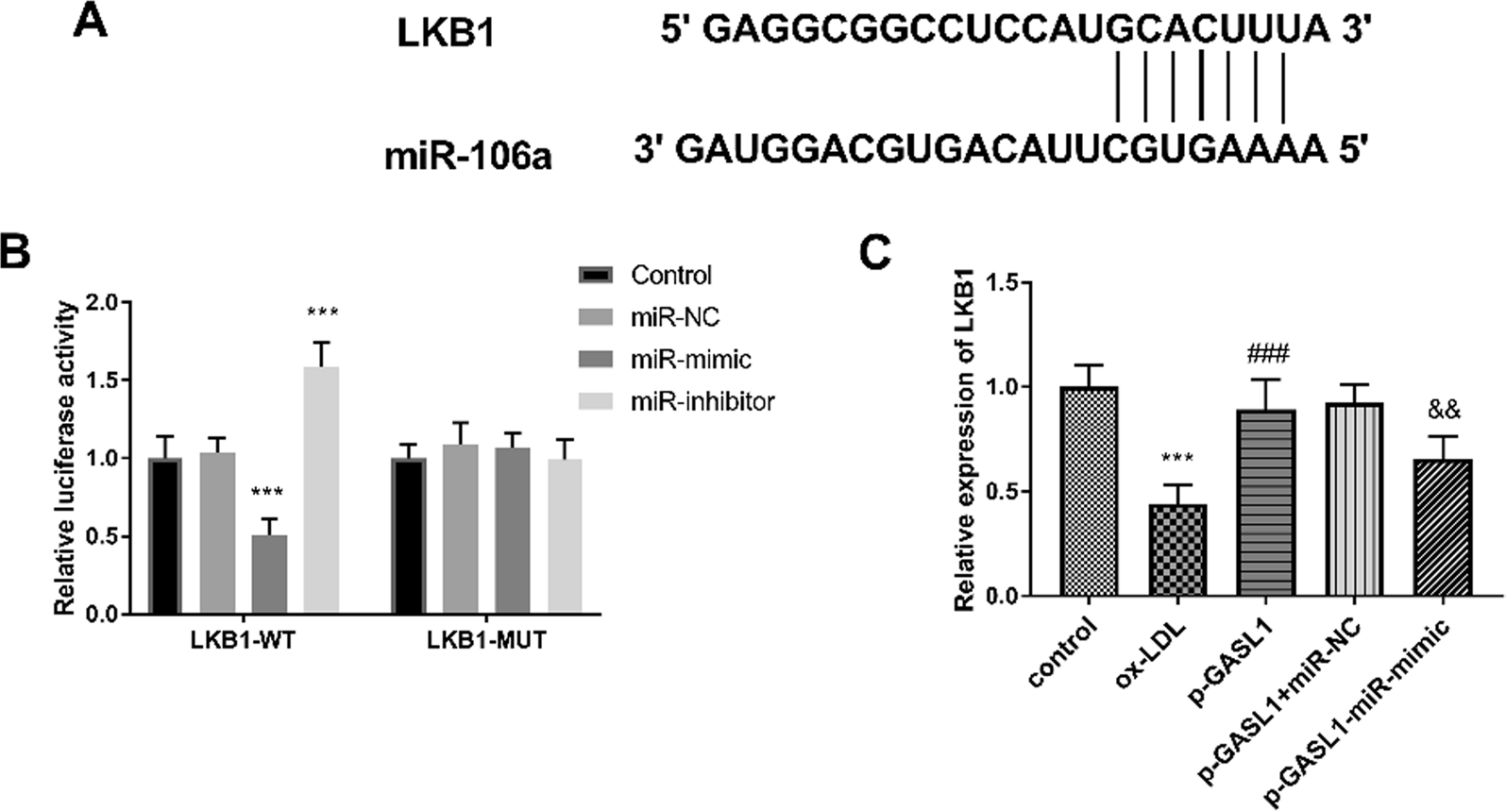
LKB1 was a direct target of miR-106a. **A** The putative binding bases between miR-106a and LKB1. **B** Luciferase report assay documented the targeted relationship between LKB1 and miR-106a. **C** The mRNA expression of LKB1 was modulated by GASL1 and miR-106a. ***P < 0.001, relative to control group; ^###^*P* < 0.001, relative to ox-LDL group; ^&&^*P* < 0.01, relative to p-GASL1 group

The binding sites between GASL1 3′UTR and miR-106a were shown in Fig. 6A. The luciferase activity of HUVECs cotransfected with LKB1-WT and miR-106a mimics was declined compared with the control group (Fig. 6B, P < 0.001). The luciferase activity of HUVECs cotransfected with LKB1-WT and miR-106a inhibitors was increased compared with control group (Fig. 6B, P < 0.001). The mRNA level of LKB1 was reduced in the ox-LDL group and enforced GASL1 led to the promotion of LKB1 expression (Fig. 6C, P < 0.001). The miR-106a intervened in the role of GASL1 on LKB1 expression (Fig. 6C, P < 0.01).

## Discussion

Atherosclerotic disease is a genetically susceptible vascular disease, which is the main cause of death and a common pathological basis of many vascular diseases [13]. The pathological process of AS involves many biological functions such as proliferation and apoptosis of important functional cells [14]. Vascular endothelial cells are the protective barrier of vascular intima, and their damage or dysfunction can lead to an increase in intimal permeability, intimal dysfunction, and AS [15]. Although the mechanism of AS has been deeply studied, as a genetic susceptibility disease, the research on transcriptome level can fully explain the pathogenesis and provide opportunities for developing new biomarkers and treatments [16]. Thus, it is of great significance to analyze the influencing factors and pathogenesis of AS.

LncRNA has become a hot topic in the field of scientific research and attracted the attention of many researchers. With the in-depth study of lncRNA, it is found that lncRNA is involved in the occurrence and development of AS [17]. Bai and colleagues certify that silenced lncRNA AK136714 inhibits atherosclerosis by down-regulating inflammatory responses and promoting apoptosis of HUVECs [18]. As a common lncRNA, the values of GASL1 are researched in several vascular disorders. In patients with intracranial aneurysms, the levels of GASL1 are declined, indicating the correlation between GASL1 and vascular diseases [19]. In the current investigation, we assess the expression of GASL1 in AS patients. Our finding indicated that GASL1 was lowly expressed in AS group, suggesting AS might contribute to the alternation of GASL1 expression. In addition, the ROC curve was drawn to interpret that GASL1 could differentiate AS patients from healthy people with high accuracy.

Vascular endothelial cells are very important for regulating vascular homeostasis [20]. Endothelial cell apoptosis is the main form of endothelial injury and plays an important role in the occurrence and development of AS [21]. Ox-LDL can lead to endothelial cell injury and promote the occurrence and development of AS [22]. The results also identified this phenomenon, that is, 50 µg/ml ox-LDL caused the decreased viability of HUVECs. Moreover, we found that the treatment of ox-LDL led to the lessened expression of GASL1, which was consistent with the finding in patients with AS. A number of lncRNAs have verified the role of endothelial cells. The treatment of ox-LDL can induce the dysfunction of HUVECs, which is further regulated by lncRNA XIST [23]. LncRNA HOXA-AS3 promotes the progression of AS through the mediation of miR-455-5p/p27 Kip1 [24]. The upregulation of GASL1 ameliorated cell viability, apoptosis, and inflammation caused by ox-LDL, thus, inhibiting the development of AS.

A large number of studies have found that ncRNA regulates the differentiation of HUVECs at the post-transcriptional level and affects the proliferation and migration of HUVECs. The ceRNA of GASL1 was tested. The finding of luciferase activity report certified that miR-106a was a sponge of GASL1. In gastric carcinoma, GASL1 is identified as a regulator of miR-106a [25]. The expression of miR-106a was elevated in HUVECs treated by ox-LDL, and it mediated the function of GASL1 on the survival rate and inflammatory situation of HUVECs. In another article on AS, the expression of miR-106a is upregulated in cells treated by ox-LDL and it participates in the activity of cells [26]. The level of miR-126 is elevated in the tissues and blood samples of patients with AS [27]. Additionally, LKB1 was tested as a directly targeted gene of miR-106a. The expression of LKB1 in HUVECs treated by ox-LDL was decreased, and its expression was regulated by GASL1 and miR-106a, suggesting the GASL1/miR-106a/LKB1 axis might play significant roles in AS. Declined LKB1 level in AS aggravates vascular damage by promoting macrophage dysfunction and the formation of foam cells [28, 29]. A small human sample size was a limitation of this study.

Taken together, the expression of GASL1 was abnormally decreased in AS patients and HUVECs induced by ox-LDL. Lessened expression of GASL1 might be a biomarker focusing on distinguishing AS patients. GASL1 militated AS development by promoting cell viability, inhibiting cell apoptosis, and silencing inflammatory responses via sponging miR-106a. LKB1 and miR-106a had a targeted relationship in AS.

## Data Availability

The data and materials used and analysed during the current study are available from the corresponding author on reasonable request.
